# The Involvement of Metals in Alzheimer’s Disease Through Epigenetic Mechanisms

**DOI:** 10.3389/fgene.2020.614666

**Published:** 2020-12-08

**Authors:** Menghua Cai, Xiangjin Zhang, Wei He, Jianmin Zhang

**Affiliations:** State Key Laboratory of Medical Molecular Biology, Department of Immunology, Institute of Basic Medical Sciences, Chinese Academy of Medical Sciences, Beijing, China

**Keywords:** Alzheimer’s disease, DNA methylation, histone modifications, metals, demenita

## Abstract

Alzheimer’s disease (AD) is the most frequent cause of dementia among neurodegenerative diseases. Two factors were hypothesized to be involved in the pathogenesis of AD, namely beta-amyloid cascade and tauopathy. At present, accumulating evidence suggest that epigenetics may be the missing linkage between genes and environment factors, providing possible clues to understand the etiology of the development of AD. In this article, we focus on DNA methylation and histone modification involved in AD and the environment factor of heavy metals’ contribution to AD, especially epigenetic mechanisms. If we can integrate information together, and that may find new potential targets for the treatment.

## Introduction

Neurodegenerative disorders are characterized by the progressive accumulation of misfolded proteins, which trigger damage of synapses, disturb network of pathway, facilitate death of specific neuronal populations, and finally initiate diseases. Several factors were hypothesized to be associated with the etiology of those diseases, including genetic and environmental factors. Alzheimer’s disease (AD) is the most common neurodegenerative disease, and the hallmarks of AD pathology are an accumulation of A*β* to form amyloid-plaques and aggregation of phosphorylated tau to constitute neurofibrillary tangles (NFTs). A*β* is viewed as the core stone and trigger of diseases, which induces the dysfunction of synapses, loss of neurons, and ultimately dementia, with the existence of A*β* plaques and NTFs (Morris et al., 2014). Hyper-phosphorylation changed the conformation of tau, which was believed to play a role in synaptic plasticity and facilitated its misfolding in pathological process (Zhang et al., 2016). Beside, apolipoprotein E (*ApoE*) gene shows strong association with risk for AD, for ApoE combined directly with A*β* to promote its aggregation and that facilitated tau phosphorylation inducing NFTs (Brecht et al., 2004).

Epigenetics is the study of heritable and reversible changes in gene expression, including DNA methylation, multi-modification of histones, and microRNA (Collotta et al., 2013), which occur without a change in the DNA sequence. This article reviewed DNA methylation and histone modifications to exhibit latest understanding about the role epigenetics plays in AD.

### DNA Methylation

The first report epigenetic changes in AD found hypomethylation of *amyloid precursor protein* (*APP*) from an AD patient (West et al., 1995). In a pair of monozygotic twins, levels of DNA methylation significantly decreased in temporal neocortex neuronal nuclei of the AD twin (Mastroeni et al., 2009). Besides, DNA methyltransferase (DNMT) decreased in entorhinal cortex layer II of AD patients (Mastroeni et al., 2010). In a recent research, the patient group showed 25% reduction of DNA methylation levels in mitochondrial DNA D-loop region (Stoccoro et al., 2017), suggesting the underlying role of mitochondrial DNA methylation in AD. Hypomethylation of *BRCA1* was observed in AD patients, and this result was in consistent with the higher expression of its mRNA (Mano et al., 2017). Through comparing brains of mouse models and AD patients, hyper-methylation of three genes namely TBXA2R, SPTBN4, and SORBS3 resulted in silence of these genes in AD process (Sanchez-Mut et al., 2013).

### Histone Modification

Comparing the temporal cortex and hippocampus, the twin with AD showed a significantly higher level of H3K9me3, a sign of gene silence, and H3S10 phosphorylation, a regulator of chromatin structure (Wang et al., 2013). The brains from AD patients showed hyper-acetylation in histone H3 and H4 (Narayan et al., 2015). Histone deacetylation catalyzed by histone deacetylase (HDAC) results in a condensed state of chromatin and consequent transcriptional repression. HDAC2 increased in AD-related neurotoxic insults *in vitro*, two mouse models and patients with AD, which decreased the histone acetylation of genes related to memory and inhibited their expression (Graff et al., 2012). Tau interacts with HDAC6 to decrease its activity. Through this way, tau promoted the acetylation of related genes (Perez et al., 2009). As a feedback and compensation, the expression of HDAC6 was significantly increased. In a mouse model of AD, decreased HDAC6 facilitated the recovery of learning and memory through disturbing mitochondrial trafficking dysfunction caused by A*β* (Govindarajan et al., 2013). Importantly, the AD mouse model treatment with valproic acid (VPA), one of widely used HDAC inhibitors in clinical research, has shown exciting results. VPA significantly decreased Aβ production by inhibiting *γ*-secretase cleavage of APP and alleviated the memory deficits of the AD mice (Qing et al., 2008).

### Roles of Metals in AD

#### Plumbum

Plumbum facilitated the concentrations of free radicals, which leaded to the death of neurons. Pb exposure stimulated the serine/threonine phosphatases to impair memory formation (Rahman et al., 2011). Pb exposure leaded to the DNA methylation changes in the whole blood cells (Hanna et al., 2012). Early exposure of Pb increased A*β* product in old age. While in aged monkeys exposed to Pb as infants, the expression of APP and BACE1 elevated, and the activity of DNMT decreased (Wu et al., 2008). In rodents exposed lead, the expression of APP increased 20 months later, implying that lead exposure showed a life-long risk of AD (Basha et al., 2005).

In mice model of AD exposure to Pb, the levels of DNMT1, H3K9ac, and H3K4me2 decreased, the level of H3K27me3 increased, while the concentration of DNMT3a did not change (Eid et al., 2016). Besides, Pb exposure altered the production of tau (Dash et al., 2016). In mice expressing human APP, Pb stimulated the production of A*β* (Gu et al., 2011). Pb also disturbed the clearance of Aβ plaques by suppressing the activity of neprilysin (Huang et al., 2011). In primates with early exposure of Pb, their brains showed overexpression of APP and Aβ through hypo-methylation of related genes when aging. Yegambaram also reported that early exposure of Pb leaded to overexpression of APP, BACE1, and PS1, one of their regulators (Yegambaram et al., 2015). Both of them suggest that early exposure of Pb played a role in the development of AD when aging.

#### Arsenic

S-adenosyl-methionine (SAM) is essential for methylation of inorganic arsenic to detoxication, and it is also the metyl-donor required by DNA methyltransferases. So, it is reasonable to speculate that arsenic exposure leads to hypo-methylation of DNA and facilitates tumor-related gene expression (Zhao et al., 1997). Insufficiency of SAM leaded to hypomethylation of *PS1* and *BACE* genes. This hypomethylation increased the expression of PS1 and BACE, which facilitated the production of A*β* (Fuso et al., 2005). Besides, arsenic inhibited the expression of the DNA methyltransferase genes, DNMT1 and DNMT3a (Reichard et al., 2007). Sodium arsenite exposure inhibited HDAC p300 for attenuating H3K27ac at enhancers in mouse embryonic fibroblast cells (Zhu et al., 2018). Su reported a dose-response relationship between the environmental concentration of total arsenic in topsoils and the prevalence and mortality of AD in European countries (Yegambaram et al., 2015).

Environmental toxin arsenite induced a remarked increase in the phosphorylation of several sits in tau, including Thr-181, Ser-202, Thr-205, Thr-231, Ser-262, Ser-356, Ser-396, and Ser-404, which was in coincidence with results from AD (Giasson et al., 2002). Gong argued that arsenic stimulated the generation of free radicals, which leaded to oxidative stress and neuronal death (Gong and O’Bryant, 2010). When mothers were exposed to arsenic during pregnancy, their children showed a higher activation of inflammation-related pathways involved in the development of AD (Fry et al., 2007).

#### Aluminum

Aluminum has been reported to induce neurofibrillary degeneration in neurons of higher mammals in 1970s (Crapper et al., 1973). McLachlan reported a dose-effect association between the risk of AD and residual aluminum in municipal drinking water. The estimated relative risk of AD for residents with drinking water containing more than 100 ug/L of Al was 1.7 (McLachlan et al., 1996). Walton (2014) reported that long term intake of Al was an etiology of AD. A 15-year follow-up implemented by Rondeau et al. (2009) also showed a significant association between a high daily intake of aluminum and increased risk of dementia. Al could selectively interact with A*β* to facilitate the formation of fibrillar aggregation, while copper, iron, or zinc could not (Bolognin et al., 2011).

In transgenic mice overexpressed human APP (Tg2576), dietary Al stimulated the expression and aggregation of Aβ through increasing oxidative stress (Pratico et al., 2002). In embryo rat hippocampal neurons, high concentration of Al facilitated the production of ROS induced by Fe (Xie et al., 1996). Al facilitated the degradation from APP to the aggregation of Aβ (Kawahara et al., 1994). Besides, the structure of non-Aβ component of AD amyloid was changed by the induction of Al to resist degradation and form plaque (Paik et al., 1997).

## Conclusion

No mutation in genes has been definitely associated with neurodegenerative diseases, suggesting that, besides risk factors of gene, environmental exposure also is involved in the etiology of AD, and those two factors may be abridged through epigenetic alterations. Recently, an integrated multi-omics analyses identified molecular pathways associated with AD and revealed the H3 modifications H3K27ac and H3K9ac as potential epigenetic drivers linked to transcription and chromatin and disease pathways in AD (Nativio et al., 2020). These findings provide mechanistic insights on AD for aiming epigenetic regulation of therapeutic strategy. We should get more enlightenment from it and explore the relationship between AD and epigenetics. On this basis, we will further study the effective diagnosis, treatment, and prevention methods of AD, and develop new intervention measures for AD from the field of epigenetics.

## Author Contributions

MC wrote the manuscript. XZ helped to edit the manuscript. WH and JZ revised the manuscript. All authors contributed to the article and approved the submitted version.

### Conflict of Interest

The authors declare that the research was conducted in the absence of any commercial or financial relationships that could be construed as a potential conflict of interest.
